# Enhancing the Performance and Photostability of Perovskite Solar Cells with a Multifunctional Light‐Management Composite

**DOI:** 10.1002/smsc.202500330

**Published:** 2025-10-02

**Authors:** Seyede Maryam Mousavi, Mostafa Othman, Fanxing Zou, Noora Lamminen, Girish Tewari, Hossein Baniasadi, Pedro Silva, Yujiao Dong, Janne Halme, Aïcha Hessler‐Wyser, Christian M. Wolff, Paola Vivo, Muhammad Imran Asghar, Jaana Vapaavuori

**Affiliations:** ^1^ Department of Chemistry and Materials Science Aalto University School of Chemical Engineering FI‐02150 Espoo Finland; ^2^ Institute of Electrical and Microengineering (IEM) Photovoltaics and Thin‐Film Electronics Laboratory (PV‐Lab) École Polytechnique Fédérale de Lausanne (EPFL) 2000 Neuchâtel Switzerland; ^3^ Hybrid Solar Cells Faculty of Engineering and Natural Sciences Tampere University P.O. Box 541 FI‐33014 Tampere Finland; ^4^ Polymer Synthesis Technology School of Chemical Engineering School of Science Aalto University FI‐02150 Espoo Finland; ^5^ Department of Applied Physics School of Science Aalto University FI‐02150 Espoo Finland; ^6^ Renewable Energy Technologies Group Faculty of Engineering and Natural Sciences Tampere University P.O. Box 541 FI‐33014 Tampere Finland

**Keywords:** bio‐inspired substrates, performance, perovskite solar cells, stability, UV blocking

## Abstract

A multifunctional light management layer for perovskite solar cells (PSCs) is presented, made from anisotropic pectin cryogel infiltrated with poly(methyl methacrylate), further enhanced by the incorporation of 2,2′,7,7′‐tetrabromo‐9,9′‐spirobifluorene. The effectiveness of the composite layers is evaluated by attaching them to the front glass surface of the PSCs. As a result, the current density of the functionalized PSC increases by an average of 4.4 ± 0.3% relative to pristine PSCs. The improvement is credited to the presence of haze, downconversion, and a 50% reduction in reflectance between 400 and 800 nm compared to glass. The power conversion efficiency of composite‐attached PSCs increases by 5 ± 0.2% relative to pristine PSCs. Moreover, the composite effectively mitigated UV‐induced photodegradation and localized heating, extending the operational stability of PSCs, as proven by maximum power point tracking tests. The surface temperature decreases, and the T_80_ of the functionalized PSCs increases by up to 2.6‐fold compared to pristine PSCs, primarily due to the composites’ significantly low thermal conductivity and UV blocking. These findings suggest that this eco‐friendly and lightweight composite offers a viable solution for better‐performing and more stable PSCs, advancing the potential for their widespread commercial adoption in various environments, including heavy UV exposure.

## Graphical abstract

The glass substrate in perovskite solar cells (PSC) causes high reflection, transmits UV light, and conducts heat, accelerating degradation. A multifunctional pectin/PMMA–spirobifluorene composite with haze, UV absorption and downconversion, and heat dissipation is introduced. Optically coupling it to PSCs enhances efficiency and stability, paving the way for the fabrication of eco‐friendly, UV‐protective PSC substrates.

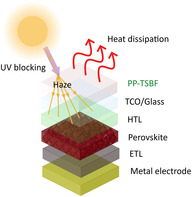

## 1 Introduction

The rapid advancement of perovskite solar cells (PSCs) has shaken up the photovoltaic landscape, owing to their high‐power conversion efficiencies (PCEs) and low production costs. However, for PSCs and rival traditional silicon‐based solar technologies alike, optimizing their light management capabilities is crucial to improve performance and longevity.^[^ ^1^, ^2^, ^3^ ^]^ Effective control of optical properties, such as reflection and haze, at the interface between PSCs and air, can significantly enhance light absorption and device efficiency.^[^ ^4^ ^]^ For instance, introducing haze by textured coatings can scatter light in multiple directions, cut reflection, and thereby boost absorption and PCE.^[^ ^5^, ^6^, ^7^ ^]^ Additionally, the sun's spectrum contains high‐energy UV radiation, which is divided into UVC (100–275 nm), UVB (275–320 nm), and UVA (320–400 nm) regions.^[^ ^8^, ^9^, ^10^ ^]^ Prolonged exposure to UVA and UVB, which cannot be absorbed by the Earth's atmosphere, can cause the dissociation of molecular components and ion migration within the perovskite layer, ultimately decreasing the performance and lifespan of PSCs.^[^ ^11^, ^12^, ^13^, ^14^ ^]^ This issue is even more pronounced in extreme environments like outer space, where intense UV radiation is a significant concern.^[^ ^15^, ^16^, ^17^ ^]^ Therefore, the effective capture and utilization of sunlight, along with the mitigation of harmful UV radiation and thermal effects, are critical for enhancing the durability of PSCs. Considering the pivotal role that light plays in both enhancing performance and maintaining stability, integrating a light management layer that simultaneously addresses these challenges can dramatically improve the overall PCE and reliability of PSCs.^[^ ^5^, ^18^ ^]^

To this end, our group has previously developed a lightweight, bio‐inspired, and partially bio‐based optically transparent composite made from pectin and poly(methyl methacrylate) (pectin/PMMA).^[^ ^19^ ^]^ Herein, we explore the hypothesis that this material can be used as a light management composite and eventually as an alternative to glass as the substrate of PSCs. This composite shows promising UVA‐blocking properties and offers higher haze than glass, which is beneficial for light scattering and enhancing absorption in PSCs.^[^ ^19^ ^]^ However, the pristine pectin/PMMA composite faces drawbacks, particularly its photothermal properties and partial UV protection, which can limit its ability to efficiently improve the performance and longevity of the solar cell. To overcome these limitations and further enhance the composite, 2,2′,7,7′‐tetrabromo‐9,9′‐spirobifluorene (TSBF) was incorporated into the PMMA matrix owing to its unique structural and optical properties, and solvent‐free miscibility with PMMA. As reported in the literature, different types of spiro‐based compounds were used to tune the porosity and the optical properties of diverse polymers, such as PMMA.^[^ ^20^, ^21^, ^22^ ^]^ For instance, Weber et al. synthesized aromatic polyamides and polyimides using spirobifluorene monomers to achieve intrinsic microporosity, which is essential for optimizing the optical properties, surface functionality, and physical properties of the materials. In their work, the spiro structure introduced a rigid, nonplanar geometry that prevented aggregation and crystallization within the polymer matrix by providing further free volume.^[^ ^23^ ^]^ This made the TSBF a promising candidate for us to be incorporated into the PMMA structure to tune the optical properties of the final pectin/PMMA composite. Additionally, TSBF exhibits excellent UV blocking by absorbing wavelengths <390 nm and converting them to higher wavelengths at around 420 nm, which could protect the perovskite layer from harmful radiation.^[^ ^22^ ^]^

Herein, a new multifunctional composite is introduced, which acts as a heat and light management layer for the solar cells. The TSBF‐incorporated optically transparent pectin/PMMA composites successfully improved the short current density (*J*_sc_) of the PSC devices by 4.4 ± 0.3% and reduced the reflection at the air/glass interface by 50% between 400 and 800 nm. Furthermore, the composite absorbs UV light <400 nm and protects the PSC devices from the UV‐induced photodegradation. Its inherent light management capabilities, combined with bio‐based nature of pectin and reduced weight, offer significant performance, manufacturing, and sustainability advantages. The development of this composite paves the way for further innovations in light‐managing substrates and the future design of high‐efficiency, durable, and eco‐friendly perovskite solar cell substrates suitable for use in various environments.

## 2 Results and Discussion

**Figure 1:**
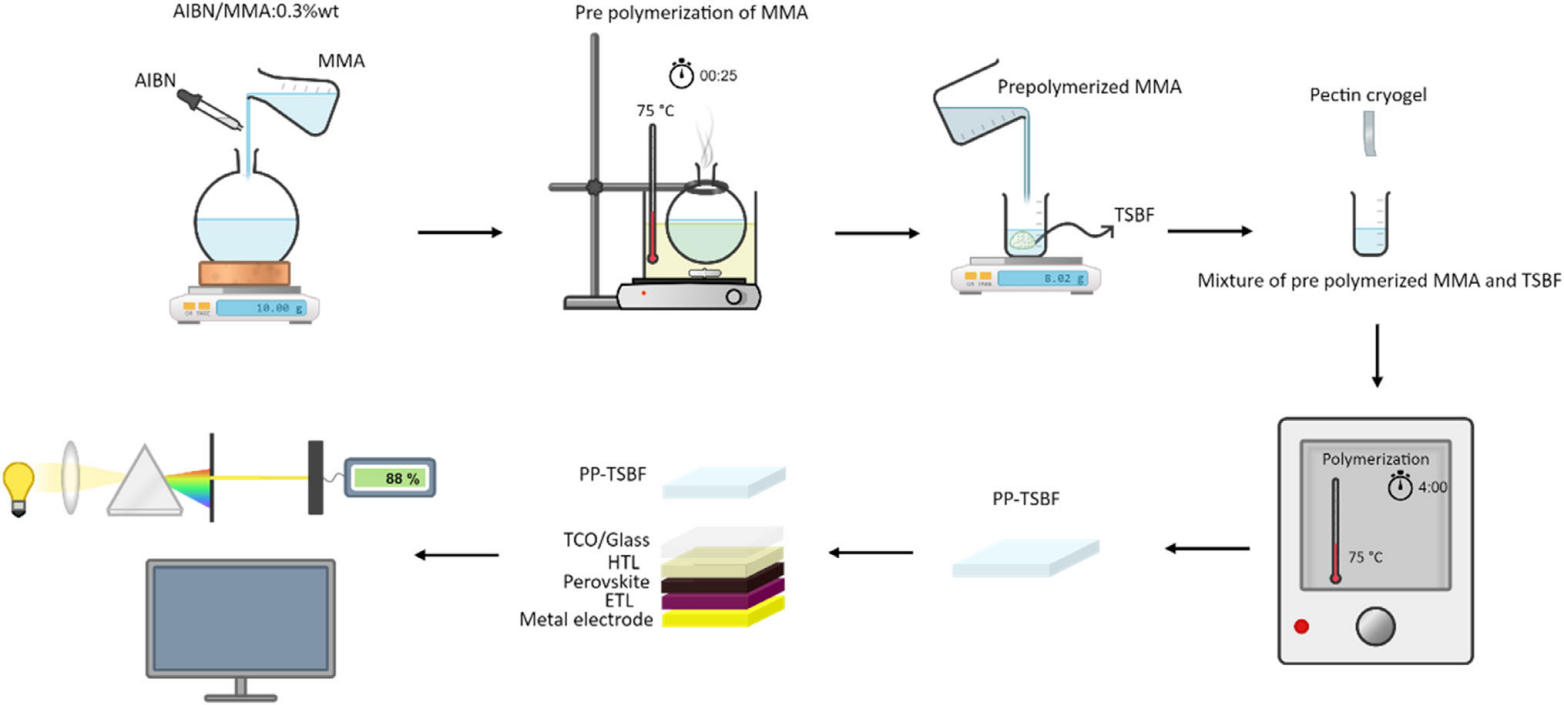
Process flowchart of fabrication of the light management layers and their attachment to the PSCs.

**Figure** **1** illustrates the experimental process for this research, which aims at enhancing the optical properties of the front surface of solar cells—crucial for maximizing the conversion efficiency of incoming light. The process starts with the mixing of methylmethacrylate monomers (MMA) and AIBN, followed by heating the mixture to 75 °C for 25 min to facilitate prepolymerization. Next, TSBF is added to the prepolymerized MMA in varying concentrations of 0, 0.25, and 0.75 wt%. These mixtures are then infiltrated into pectin cryogels to form three different composites with a thickness of 1mm: the pectin‐PMMA composite (PP composite), the pectin‐PMMA + 0.25 wt% TSBF composite (PP‐0.25TSBF), and the pectin‐PMMA + 0.75 wt% TSBF composite (PP‐0.75TSBF).

The resulting composites underwent optical tests and were then affixed onto the glass surface of the ITO/FTO in the PSCs using epoxy resin for the subsequent testing. The objective was to create a front surface layer that minimizes optical losses and scatters incoming light, allowing solar cells to utilize photon energy more efficiently. Additionally, the light management layer aims to mitigate the harmful effects of high‐energy UV radiation through downconversion.^[^ ^8^, ^24^, ^25^ ^]^

**Figure 2:**
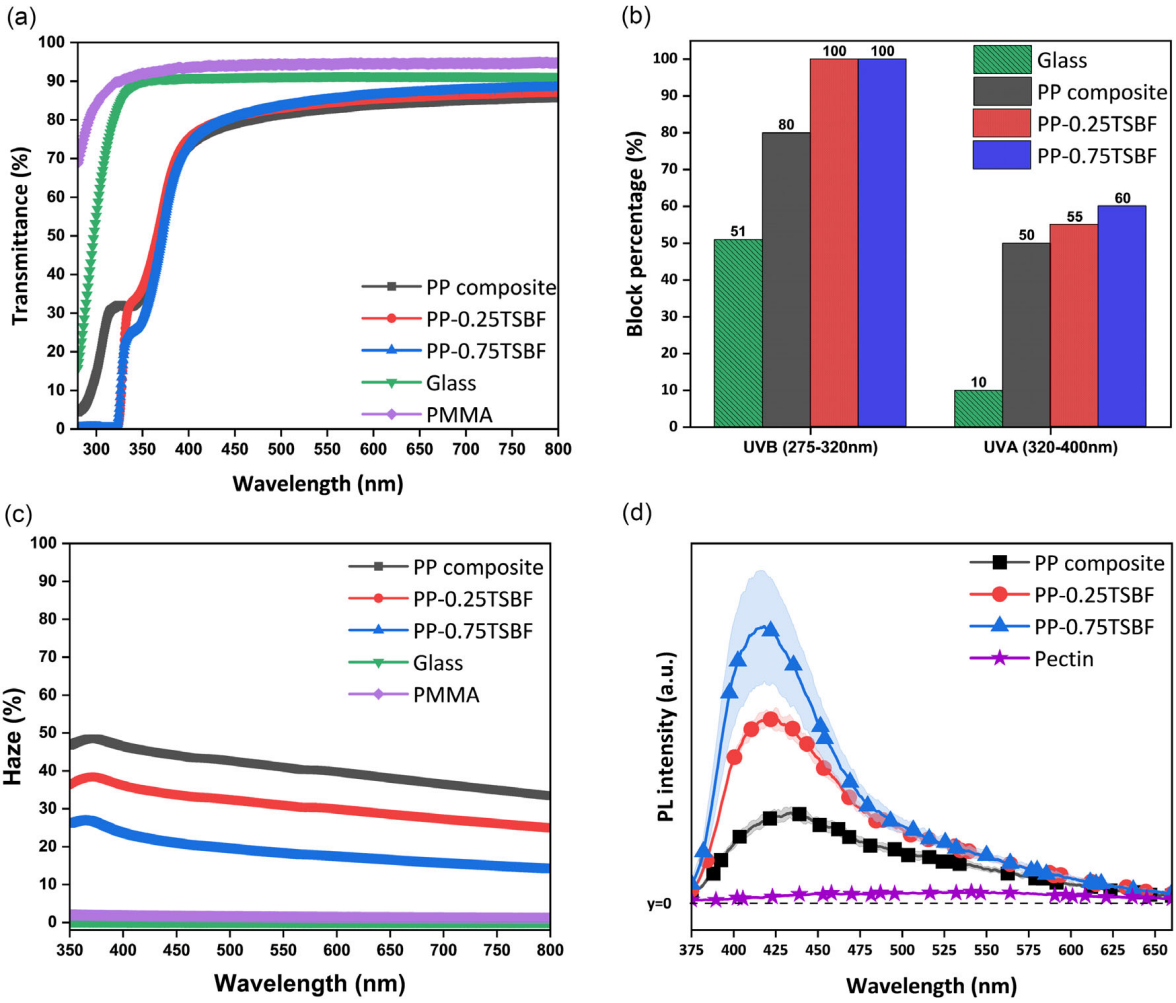
a) Transmittance, b) UV blocking calculated from the transmittance spectra, c) haze, and d) photoluminescence spectra (excitation wavelength of 350 nm) of the PMMA, PP composite, PP‐0.25TSBF, and PP‐0.75TSBF composites. Measurements were taken from three different spots on each sample (*n* = 3). Solid lines represent the average values, and shaded bands indicate the standard deviation (SD) calculated from these measurements.

**Figure** **2** presents the results of the optical measurements, namely, transmittance, UV block percentage, haze, and photoluminescence (PL) intensity. The thickness of all samples is 1 mm and this was kept constant for all the tests. The selected thickness was the lower manageable experimental limit of making such composites in our process.^[^ ^19^ ^]^ According to Figure 2a, glass and PMMA demonstrated 90% and 94% transparency, respectively, at 550 nm. The transmittance of the PP composite at 550 nm is 82%, and this value increased to 84% and 85% for the PP‐0.25TSBF and PP‐0.75TSBF composites, respectively. The UV‐blocking property of each composite was calculated from the transmittance spectra (Figure 2a) and is presented in Figure 2b. As depicted in Figure 2b, PP‐0.25TSBF and PP‐0.75TSBF composites completely blocked UVB rays from the incoming light spectrum. In contrast, the PP composite provided 80% UVB Blockage, and the glass blocked only 50% of the UVB light. The addition of TSBF also enhanced the composites’ UVA‐blocking capability. For instance, while the UVA‐blocking ability of glass is only 10%, the PP composite provided 50% shielding against UVA. Upon the addition of TSBF, the UVA blocking ability increased to 55% and 60% in PP‐0.25TSBF and PP‐0.75TSBF, respectively. Additionally, according to Figure 2c, the PP composite exhibited 41% haze at 550 nm, which decreased with the incorporation of TSBF. At this wavelength, the haze values were 31% for PP‐0.25TSBF and 18% for PP‐0.75TSBF, both of which remain higher than the nearly zero haze observed for glass and PMMA.

The photoluminescence (PL) spectra of the composites were recorded by exciting the composites with 350 nm light. As is evident in Figure 2d, all the composites demonstrated PL emission under that excitation. Looking at the spatial homogeneity of the PL spectra across the tested layer, the PL spectra of PP‐0.75TSBF exhibit a wider spread, likely due to the limited solubility of TSBF powder in prepolymerized MMA, as prepolymerized MMA and TSBF were mixed without an additional solvent. Consequently, the TSBF may not have dissolved homogeneously in the prepolymerized MMA, resulting in agglomeration within the polymer and leading to variations in the PL measurements across different spots in the composite. While the emission from the PP‐0.25TSBF and PP‐0.75TSBF composites arises from the photoluminescent nature of the TSBF,^[^ ^22^ ^]^ the emission source for the PP composite was unknown. Therefore, a series of PL tests was carried out on the optically transparent composites with various excitation wavelengths of 375 and 400 nm.

The results of the tests are presented in Figure S1, Supporting Information, where emission was observed in all PP composites, the PP‐0.25TSBF, and PP‐0.75TSBF samples. Then, the test was performed with the pectin cryogels in two different directions, parallel and vertical to pore walls, with an excitation wavelength of 350 nm. Interestingly, the emission was dependent on the orientation of the cryogels during the test. As can be seen in Figure S2, Supporting Information, a small amount of emission is detected when the test is performed parallel to pectin pore walls, while when vertically placed, no such effect was observed. This is in line with the anisotropy of the pectin cryogels and proves to be due to the presence of the pectin cryogels in the composite. Since haze originates from the anisotropy of pectin cryogels in the PP composite, we believe the PP composite traps the incident rays and diffracts them in multiple wavelengths. Presumably, this also influences the PL emission of the PP‐0.25TSBF and PP‐0.75TSBF composites. Consequently, the matrix environment plays a role in determining the composites’ emission properties, as also reflected in the long spectral tails observed in Figure 2d, S1, and S2, Supporting Information, which are consistent with those of the PP composite and pectin.^[^ ^26^ ^]^

**Figure 3:**
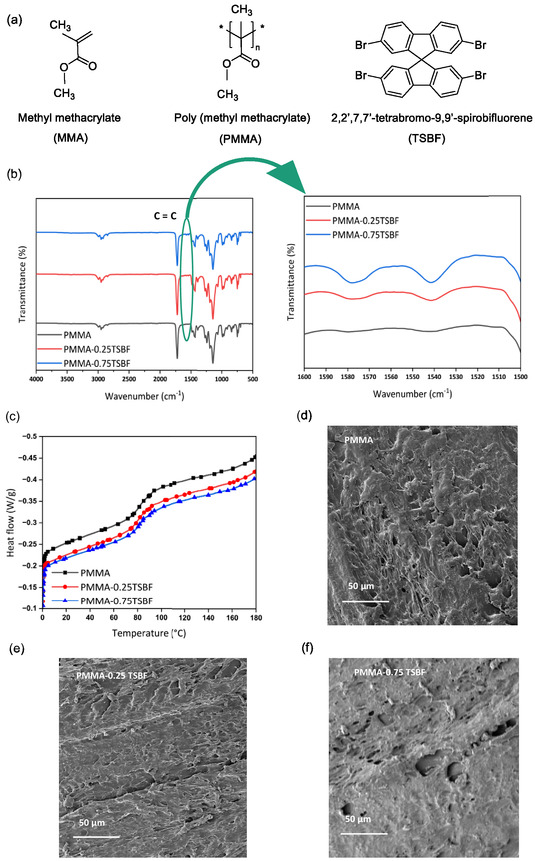
a) Chemical structures of the MMA, PMMA, and TSBF; b) FTIR spectra; c) DSC curves; and d–f) SEM images of PMMA, PMMA with 0.25 wt% TSBF(PMMA‐0.25TSBF), and PMMA with 0.75 wt% TSBF (PMMA‐0.75TSBF).

Since TSBF was added to the prepolymerized MMA before infiltration into the pectin cryogels, its interaction with the PMMA was investigated using FTIR, DSC, and scanning electron microscopy (SEM) analyses. **Figure** **3**a depicts the chemical structure of the MMA, PMMA, and the TSBF. FTIR spectra (Figure 3b) show that the characteristic peaks of PMMA are retained after doping, while two additional peaks at 1580 and 1540 cm^−1^ appear in the PMMA‐0.25TSBF and PMMA‐0.75TSBF samples. These peaks correspond to C=C stretching in the aromatic rings of TSBF, confirming its successful incorporation.^[^ ^27^, ^28^ ^]^ No significant peak shifts or new bands are observed, indicating that the interaction between PMMA and TSBF is physical rather than chemical, likely via van der Waals forces.^[^ ^29^ ^]^ DSC curves (Figure 2b) reveal a slight decrease in the glass transition temperature (*T*_g_) from 84 °C (PMMA) to 83 and 82 °C for the 0.25 and 0.75 wt% TSBF‐doped PMMA, respectively. This decrease in *T*_g_ suggests that TSBF disrupts the regular packing of PMMA chains by introducing free volume, consistent with its role as a structure‐directing additive.^[^ ^23^, ^30^ ^]^ By physically spacing out the polymer chains, TSBF hinders chain agglomeration, increases free volume, and contributes to reduced intermolecular rigidity, all of which led to enhanced optical transparency. The amorphous nature of the new composite, accompanied by further free volume, reduces scattering sites, enhancing the transmission of light through the material and contributing to increased transparency.^[^ ^31^, ^32^ ^]^ SEM images of the PMMA polymer and the PMMA‐0.25TSBF and PMMA‐0.75TSBF are present in Figure 3d. As expected, no change is visible in the morphology of the polymer films. Although no crystallinity is introduced and PMMA remains amorphous, the combination of FTIR, DSC, and SEM supports the conclusion that TSBF alters the nanoscale packing environment of the polymer, leading to structural changes that enhance optical clarity without compromising morphology.

**Figure 4:**
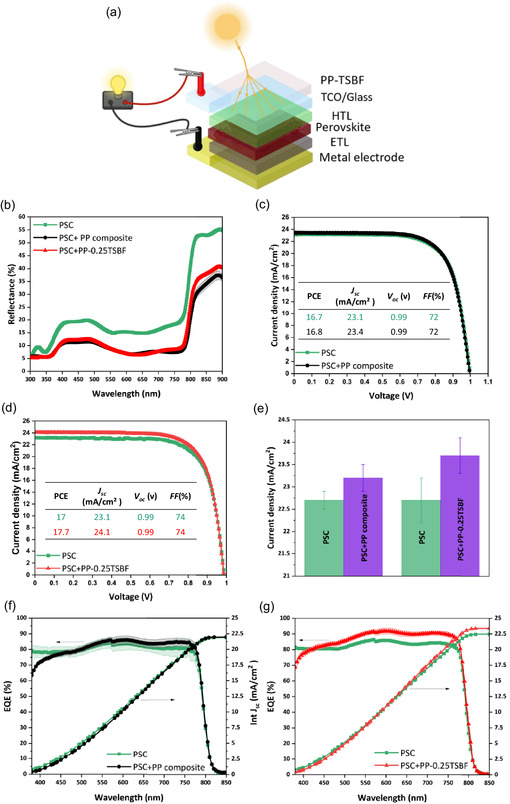
a) Schematic illustration of the proposed effect of the composites on PSCs. b) Reflection spectra of a PSC, a PSC coupled with the PP composite, and a PSC coupled with the PP‐0.25TSBF composite. *J–V* characteristics of a PSC and a PSC coupled with c) the PP composite and d) the PP‐0.25TSBF composite. e) Average *J*_sc_ values obtained from *J–V* measurements of five devices per condition (*n* = 5), with error bars representing the SD. f,g) EQE spectra of PSCs with and without the (f) PP composite and (g) PP‐0.25TSBF composite, with integrated *J*_sc_ values. Solid lines represent the average of three independent measurements (*n* = 3), and shaded regions indicate the SD. Integrated *J*_sc_ values were calculated from these averaged spectra and plotted alongside the corresponding EQE curve.

The influence of the composites’ optical properties on PSC performance was evaluated by optically coupling them to the devices. **Figure** **4**a illustrates a schematic of a composite‐integrated solar cell with a p‐i‐n architecture (glass/ITO/MeO‐2PACz/perovskite/C60/SnO_2_/Cu). As shown in Figure 4b, the reflectance from the surface of the solar cells decreases from ≈15% for the pristine devices to ≈8% for both the PP composite‐ and PP‐0.25TSBF‐integrated PSCs at 550 nm, a ≈10% absolute reduction across the visible spectrum. This improvement is attributed to better refractive index matching between the composite layer and air compared to glass and air. Although the composite layers were adhered to the glass using an epoxy resin, the epoxy's refractive index (1.5–1.6) is closely matched to that of glass, effectively minimizing interfacial reflection losses from the two additional optical interfaces.

The *J–V* behavior of the fabricated cesium‐formamidinium PSC devices was evaluated in ambient conditions, under 1 sun at room temperature 25 °C, to assess the impact of the composites’ optical properties on their electrical characteristics.^[^ ^33^ ^]^ Figure 4c,d represents the *J–V* curves of the champion PSCs with and without the composites, and the photovoltaic characteristics are summarized in the insets of each graph. Interestingly, the efficiency of the pristine PSCs remained nearly unchanged upon attaching the PP composite (see Figure 4c), due to only a small increment in *J*_sc_. Although the PP composite has higher haze properties, its lower transparency and photothermal activities reduce its overall effectiveness, resulting in a minor change in efficiency through a—on average—0.5 mA cm^−^^2^ higher short *J*_sc_ as shown in Figure 4e.^[^ ^7^ ^]^ In contrast, the PP‐0.25TSBF composite‐integrated PSC devices showed a further improved *J*_sc_, as evident in Figure 4d. According to the statistics presented in Figure 4e, this improvement is mainly attributed to a 1 mA cm^−^^2^ higher *J*_sc_ in the PP‐0.25TSBF‐integrated PSCs in comparison to pristine PSCs, translating into a ≈0.7% absolute improvement in their efficiencies (see Table S1, Supporting Information). The PP‐0.75TSBF‐integrated devices did not result in significant current improvement, most likely due to their lower haze compared to the PP‐0.25TSBF composites (see Figure S3, Supporting Information).

The integrated currents calculated from the EQE data match the improvements seen in the *J–V* measurements as evident in Figure 4f,g; Table S2, Supporting Information. In the case of PP‐0.25TSBF, the UV‐blocking reduction in *J*_sc_ is overcompensated by the overall higher EQE signal in the range 400–800 nm, in line with the results from *J–V* measurements. Notably, comparison of Figure 4f,g shows that the EQE response of the PP‐0.25TSBF‐integrated devices in the UV region (380–500 nm) is higher than that of the PSC+PP composite, although it remains lower than the pristine PSC due to the intrinsic UV‐blocking nature of the composite layer. The improvement over the PP composite highlights the downconversion effect of incorporating TSBF, as confirmed by the PL data in Figure 2d. The resulting visible photons contribute to a net gain in *J*_sc_, consistent with both the integrated EQE and *J–V* measurements.

Optical measurements confirmed that the PP‐0.25TSBF composite offers downconversion, improved transparency compared to the PP composite, 31% haze, better refractive index matching with air, and 10% absolute less reflection compared to the pristine PSCs. These, in turn, increase the light path and extend effective light absorption, improving the current density as verified by both *J–V* and EQE measurements.

**Figure 5:**
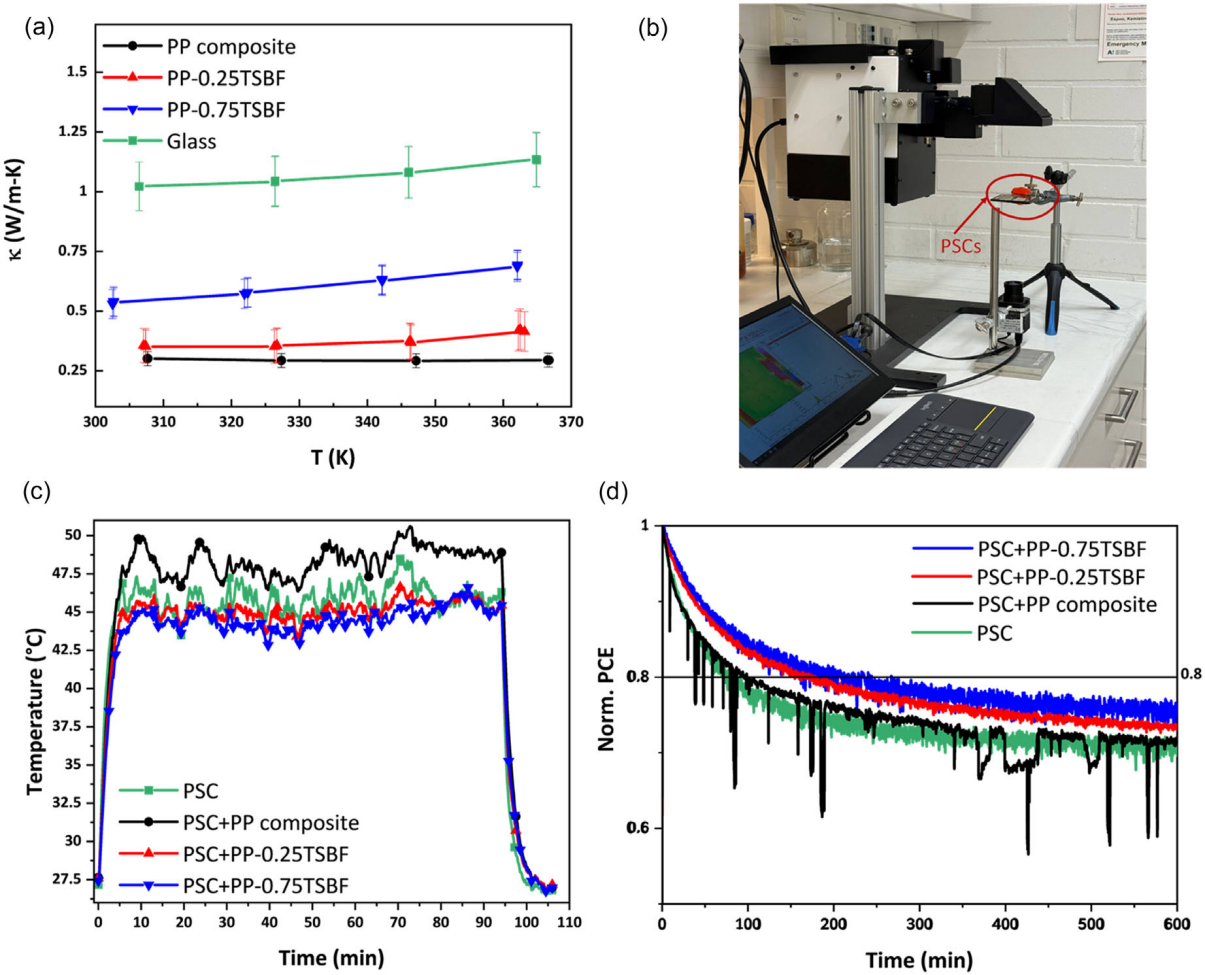
a) Thermal conductivity of glass, PP, PP‐0.25TSBF, and PP‐0.75TSBF composites. b) Set up for temperature monitoring of pristine and composite‐integrated PSCs. c) Back‐surface (gold electrode) temperature of pristine PSC and PSCs with PP composite, PP‐0.25TSBF, and PP‐0.75TSBF, under 1 sun illumination, recorded by IR camera. d) MPPT results of PSCs with and without composites; solid lines indicate T80 values.

In addition to the optical and performance enhancements, the low thermal conductivity of the composites raised the question of whether this factor could also play a role in improving thermal management in PSCs. Since temperature fluctuations during operation can influence the stability of perovskite materials, the thermal properties of the composites and their potential impact on device stability were further examined. **Figure** **5** provides a summary of the thermal properties of various composites and their impact on the photostability of PSCs. As shown in Figure 5a, all the composites have significantly lower thermal conductivity compared to glass. The PP composite has the lowest thermal conductivity, but the addition of TSBF enhances it. Specifically, the thermal conductivities for PP, PP‐0.25TSBF, and PP‐0.75TSBF composites at 33 °C are 0.30, 0.35, and 0.53 W m^−1^ K, respectively.

The low thermal conductivity of the PP composite is due to its porosity and the difference in atomic masses between its components (pectin‐PMMA), which enhances phonon scattering at the interface between the pectin pore wall and PMMA.^[^ ^19^, ^34^ ^]^ Therefore, heat dissipation in the PP composite occurs in‐plane, from the pectin pore walls to the surrounding environment, rather than vertically.^[^ ^19^ ^]^ The addition of brominated aromatic rings present in TSBF to the pectin/PMMA composite enhances physical interactions, thereby improving in‐plane phonon transport and overall thermal conductivity. This hypothesis is supported by the observed trend in the data, with the PP‐0.75TSBF composite demonstrating the highest thermal conductivity.^[^ ^35^, ^36^, ^37^ ^]^

To observe the effect of composite integration on the thermal management of PSCs, different composite‐integrated PSCs were placed under 1 sun illumination for over 100 min. An IR camera measured the temperature on the opposite side of the devices (gold electrode), as shown in Figure 5b. Figure 5c illustrates the temperature changes of PSC devices under light and after the light is turned off, with temperatures averaged from a circular area where the gold electrodes are located. The graph shows that the pristine PSC device reached maximum temperatures of around 48.5 °C, with several temperature peaks visible. Moreover, the temperature of the PP composite‐integrated device rose even higher than the temperature of the pristine PSCs due to the photothermal properties of the PP composite.^[^ ^19^, ^26^ ^]^ In contrast, coupling PP‐0.25TSBF and PP‐0.75TSBF composites to the PSCs built a more uniform device temperature profile. Furthermore, the maximum temperatures of the PP‐0.25TSBF‐ and PP‐0.75TSBF‐integrated PSCs decreased to 46.6 and 45.6 °C, respectively, demonstrating superior heat management compared to the pristine PSCs.

Maximum power point tracking (MPPT) measurements were performed under 1‐sun illumination in an N_2_ environment on pristine PSCs as well as on PSC devices attached with PP, PP‐0.25TSBF, and PP‐0.75TSBF composites to evaluate the effectiveness of the composites in improving heat management. As evident in Figure 5d, the T_80_ values improved by 1.25, 2.1, and 2.6 times for PP, PP‐0.25TSBF, and PP‐0.75TSBF attached PSCs, respectively. The initial photodegradation of PP composite‐integrated PSCs was similar to that of pristine PSCs due to heating and partial UV absorption. In contrast, PP‐0.25TSBF and PP‐0.75TSBF composites provided effective heat management and UV screening for the PSCs, thus slowing their initial photodegradation. Notably, the PP‐0.75TSBF composite demonstrated the best thermal management and highest UVA and UVB blocking, resulting in the slowest initial photodegradation. The enhanced photostability is primarily linked to the superior UVA and UVB blocking and efficient heat management due to the optimal thermal conductivity of TSBF‐infused PP composites. Incorporating TSBF into the pectin/PMMA reduces localized heat buildup by improving the in‐plane thermal energy distribution, ensuring more uniform heat dissipation rather than concentrated areas. From these observations, we infer that an optimal level of thermal conductivity is required to prevent PSC device overheating. This also depends on the composite structure, necessitating side heat‐dissipating pathways to effectively remove heat. This advanced light management layer functions as both a heat sink and a UV blocker, with the PP‐0.25TSBF and PP‐0.75TSBF composites offering the best performance in terms of enhancing the overall stability of PSC devices.

## 3 Conclusion

Overall, we developed and analyzed a novel multifunctional composite made from pectin and poly (methyl methacrylate) (PMMA), enhanced with 2,2′,7,7′‐tetrabromo‐9,9′‐spirobifluorene (TSBF), aimed at improving the light management and stability of PSCs. The inclusion of TSBF was found to enhance the composite's optical properties, including increased transparency, superior UV absorption, and improved haze, thereby facilitating better light scattering and reduced reflection losses. These improvements translated directly into enhanced solar cell performance, notably increasing *J*_sc_ by 4.4 ± 0.3% and reducing reflection by 50%, both relative to pristine PSCs. Importantly, the TSBF‐incorporated composites provided effective heat management, addressing the detrimental effects of both UV‐induced photodegradation and heating, thus extending the operational stability of the PSCs. Our findings highlight the critical role of multifunctional composites in advancing PSC technology, addressing key challenges related to light management and device longevity. The developed composite not only enhances the efficiency and stability of PSCs but also offers significant advantages in terms of manufacturing and sustainability, given their partially bio‐based origins and reduced weight. This research paves the way for the development of high‐performance, durable, and eco‐friendly perovskite solar cell substrates. The integration of such advanced materials could play a pivotal role in the future commercialization and adoption of high‐performance and durable PSCs.

## 4 Experimental Section

4.1

4.1.1

##### Materials

Pectin from citrus peel (Sigma–Aldrich), methyl methacrylate (MMA, 99%, Sigma–Aldrich), and 2,2′‐azobis(2‐methylpropionitrile) (AIBN, 98%, Sigma–Aldrich), aluminum foil (local market), fluorine‐doped tin oxide glass substrates (TEC15, FTO glass, Greatcell solar), hellmanex (Hellma), TiO_2_ paste (30 NR‐D, Dyesol), acetylacetone (P7754, Sigma–Aldrich), titanium diisopropoxide (Sigma–Aldrich), buckminsterfullerene(C60, Nano‐C, purity >99.9%), lead iodie (PbI_2_‐L0279,TCI), lead bromide (PbBr_2_‐L0288, TCI), cesium iodide (CsI, AB207757, abcr GmbH), formamidinium iodide (FAI, Greatcell), methylammoniumylamonium bromide (MABr, Greatcell solar), dimethylformamide (DMF, anhydrous, Sigma–Aldrich), dimethyl sulfoxide (DMSO, anhydrous, Sigma–Aldrich), chlorobenzene (anhydrous, Sigma–Aldrich), acetonitrile (anhydrous, Sigma–Aldrich), Me‐4PACz (TCI), Spiro‐OMETAD (Luminescence Technology Corp.), tert‐butylpyridine (tBP, Sigma–Aldrich), lithium bis(trifluoromethanesulfonyl)imide (LITFSI, Sigma–Aldrich), FK 209 Co(II) PF6 salt (Sigma–Aldrich), gold and copper pelletes (99.99%) (Kurt J. Lesker Company), and silver conductive paste (Electrolube) were used without further purification.

##### Preparation of the Light‐Managing Composites

To prepare the pectin/polymethyl methacrylate (PMMA) composite, the pectin cryogels were prepared following the recipe reported by Zou et al.^[^ ^19^ ^]^ and were cut into 2 × 2 cm pieces with a thickness of 1 mm. Then, the pectin pieces were infiltrated with prepolymerized methylmethacrylate (MMA) solution under vacuum. The prepolymerized MMA solution was made by mixing MMA and AIBN with a weight ratio of 0.3 wt%. The mixture was heated to 75 °C (in an oil bath) and kept at that temperature for 25 min. Then, the 2,2′,7,7′‐tetrabromo‐9,9′‐spirobifluorene was added to the prepolymerized MMA in 0.25 and 0.75 wt% and was stirred at room temperature for 30 min until a clear solution was achieved. Then, the solutions were infiltrated into the pectin pieces using a vacuum oven at room temperature. After the infiltration, the pectin pieces, already infiltrated with a solution of prepolymerized MMA, were placed between two aluminum foil‐covered glass slides. A 1 mm glass was put between the aluminum foil‐covered glass slides to control the thickness. The further polymerization of MMA happened in the infiltrated pectin under 75 °C in an oven for 4 h. This further polymerization processes the MMA, resulting in the creation of a pectin/PMMA (PP), pectin/PMMA + 0.25 wt% TSBF (PP‐0.25TSBF), pectin/PMMA + 0.75 wt% TSBF (PP‐0.75TSBF) composites.

##### Device Fabrication

To demonstrate the effect of the light‐managing composite on the power conversion efficiency (PCE), planar P‐I‐N PSCs were fabricated based on the following procedure.

##### P‐I‐N Fabrication Procedure

ITO glass substrates were washed with Hellmanex and then deionized water for 15 min, followed by 10 min water‐bath sonication. The cleaned substrates were treated with UV ozone (UV Ozone Cleaner – ProCleaner, Bioforce nanosciences) for 10 min. After UV ozone treatment, 0.5 mg mL^−1^ of Me‐4PACz self‐assembled monolayer (SAM) solution (hole‐transport layer) dissolved in ethanol was spin coated on substrates at 3,000 rpm for 30 s in a N2‐filled glovebox, followed by annealing at 100 °C for 10 min. Then, SiO_*x*_‐NPs (prepared in‐house) were spin‐coated and annealed to form a partial coating and improve wettability.^[^ ^38^ ^]^ The spin‐coated CsFA perovskite films were deposited and then annealed at 150 °C for 30 min.^[^ ^33^ ^]^ All the fabrication processes were done inside the glovebox. An electron‐selective stack of C60 (20 nm, >99.95%, NanoC) was then thermally evaporated in a home‐made evaporation system (base pressure <2 × 10^−6^ mbar, working pressure >3 × 10^−6^ mbar, evaporation rate of 0.3 Å s^−1^ as measured by quartz crystal monitors, aluminum oxide crucibles with power applied to the sources of ≈50 W , the substrate holder was maintained at room temperature). A buffer layer of 40 nm of SnO2 was deposited by atomic layer deposition using an Oxford Instruments system at 100 °C using tetrakis(dimethylamino) tin (STREM) and H_2_O as precursors. To finish the full device, 130 nm of Cu was thermally evaporated through a shadow mask on the samples. During all the thermal evaporations, the deposition rate was first stabilized to the targeted value before opening the substrate shutter.

##### n‐i‐p Structure Fabrication

The mesoporous structure devices were used for testing the stability of the devices under operational conditions due to their vulnerability to UV light. The solar cells were fabricated with minor changes based on the procedure reported by Saliba et al.^[^ ^39^ ^]^

The substrates were cleaned by ultrasonication in Hellmanex, ethanol, and isopropanol. To ensure the removal of impurities and improve the wettability, the substrates were treated with UV‐ozone (UV Ozone Cleaner – ProCleaner, Bioforce nanosciences). After drying in a hydrothermal oven (Hydrothermal Oven Memmert 100‐800), the TiO_2_ compact layer was deposited by spray pyrolysis of the solution containing acetylacetone (0.4 mL), titanium diisopropoxide bis(acetylacetonate) (0.26 mL) in 6 mL of ethanol at 450 °C on 20 pieces of pre‐cut FTO substrates (1.4 × 2.4 cm^2^). A mesoporous TiO_2_ (m‐TiO_2_) layer was deposited onto the c‐TiO_2_ substrate by spin coating a dispersion of 30NRD TiO_2_ paste in ethanol (150 mg mL^−1^) at 4000 rpm for 10 s, followed by annealing at 450 °C. After cooling down to room temperature, the samples were transferred to the nitrogen‐filled glovebox. In order to make the triple‐cation perovskite, first the 1.5 m PbI_2_ and PbBr_2_ stock solutions were prepared in anhydrous DMF: DMSO (4:1, v:v) and heated up to 180 °C. The FAPbI_3_ solution was prepared by adding PbI2 (1.1 m) to FAI (1.08 m) powder. The MAPbBr_3_ solution was prepared from a precursor solution containing MABr (0.22 m) and PbBr_2_ (0.24 m) powder. CsI powder was dissolved as a 1.5 m stock solution in DMSO. The precursors were then mixed in (FAPbI_3_/MAPbBr_3_/CsI) 83:17:5 volume ratios. The final precursor solution was spin coated on the mesoporous layer in two steps: 1) 10 s at 1000 rpm and 200 rpm s^−1^ and 2) 20 s at 6000 rpm and 2000 rpm s^−1^. Ten seconds before the second step finishes, 150 μL of chlorobenzene was poured on the spinning substrates. Then, the substrates were annealed for 45 min at 100 °C. To form the hole transport layer (HTL), 50 μL of the HTL solution reported elsewhere^[^ ^29^ ^]^ was dropped onto prepared PSK films and spin coated at 4000 rpm for 10 s. Then, 80 nm of gold was thermally evaporated (Moorfield minilab 090) on the HTL layers. To ensure a better electrical connection, the electrodes were painted with silver paste.

##### Current–Voltage Measurement

The current (*I*)–voltage (*V*) characteristics of the cells were measured using a Keithley 2401 source measure unit under air mass (AM) 1.5 G simulated sunlight (100 mW cm^−2^) at 25 °C.

##### External Quantum Efficiency Measurement

The external quantum efficiency (EQE) of the PSC devices was measured using a solar cell spectral response measurement system (QEX7, Serial #81) (PV Measurements, Inc.).

##### Maximum Power Point Tracking

The MPPT test was carried out using the Litos Lite parallel JV system (FLUXiM AG, Switzerland) under AM 1.5 G simulated sunlight (1‐Sun, 100 mW cm^−2^), which was obtained with an A++A+A solar simulator (Sinus‐70 LED simulator from Wavelabs, Germany) and N_2_ atmosphere. MPPT was performed under continuous 1‐Sun illumination.

##### Optical Property Measurement

The optical characteristics of the composites were recorded using a UV‐2600 UV‐vis spectrophotometer integrated with an ISR‐2600Plus integrating sphere attachment (Shimadzu, Japan). The total light transmittance, haze, and reflectance were measured for the wavelength range of 250–800 nm. The average and standard deviations were calculated from 3 sets of measurements. The transmittance and haze of the composites were calculated using the following equations.

(1) $$\text{Transmittance }\left(\%\right)\;=\;T_{2}/T_{1}\times \;100\%$$

(2) $$\text{Haze }\left(\%\right)=\left(T_{4}/T_{2}-\;T_{3}/T_{1}\right)\times 100\%$$

*T*_1_, *T*_2_, *T*_3_, and *T*_4_ represent the following: the reference transmittance measured without a sample, the transmitted light intensity measured through the sample, the beam scattering measured without a sample, and the diffusive transmittance measured through the sample, respectively.

UV blocking was calculated from the transmittance spectra using the following equations

(3) $$UVA\;blocking=100\%-\frac{\int_{320}^{400}T(\lambda )d\lambda }{\int_{320}^{400}d\lambda }$$

(4) $$UVB\;blocking=100\%-\frac{\int_{275}^{320}T(\lambda )d\lambda }{\int_{275}^{320}d\lambda }$$

The reflection from the surface of PSCs, with and without composites, was calculated using the following equation

(5) $$\text{Reflectance }\left(\%\right)\;=\;R_{2}/R_{1}\times \;100\%$$

*R*_1_ and *R*_2_ indicate the reflectance measured with the white background and the measured reflectance in the presence of the sample, respectively.

##### Morphology Imaging

A scanning electron microscope (Tescan Mira3) was utilized to examine the composites’ cross‐sectional structure. The samples were first immersed in liquid nitrogen and then cut. Afterward, they were sputter‐coated with a 5 nm layer of Au/Pd (80/20) using a Q150 T coater (Quorum).

##### Photoluminescence Measurement

The photoluminescence spectra of the composites were recorded by using a PerkinElmer LS 55 fluorescence spectrometer at exciting wavelengths of 350, 375, and 400 nm.

##### Fourier Transform Infrared

Fourier transform infrared (FTIR) spectroscopy was conducted to analyze the chemical structure and functional groups of the samples. FTIR measurements were performed using a PerkinElmer FTIR spectrometer equipped with an attenuated total reflectance accessory. The spectra were recorded in the range of 4000–500 cm^−1^, with a resolution of 4 cm^−1^, and 16 scans were averaged to improve the signal‐to‐noise ratio. All spectra were obtained at room temperature.

##### Thermal Conductivity Measurement

The thermal conductivity of the composites was measured using the Thermal Transport Option (TTO) puck available in the Quantum Design PPMS. A rectangular sample (≈4–5 × 3–4 × 1–3 mm) was attached between two gold‐plated copper electrodes using thermally conductive glue. A heater and a thermometer were attached to one electrode, while another thermometer was attached to the second electrode, which was also connected to a heat sink. Under isothermal conditions, the heater applied heat to one end of the sample, and the temperature difference was measured at a steady state. The thermal conductivity was then estimated based on the sample dimensions and the measured temperature difference.

##### IR Imaging

Samples were placed under a solar simulator (model 11002 SunLiteTM, Abet Technologies), and temperature changes were monitored using a thermal camera (PIR uc 605, Infratec) with a wide‐angle lens and an image resolution of 640 × 480 pixels). The lamp of the sun simulator was turned on for 30 min before running the experiments. After the warm‐up period, the system was calibrated to register the value of 1 sun in the center of the irradiation area. The thermal camera was placed ≈15 cm under the samples, and the focus was adjusted manually. The experiment consisted of illuminating the samples continuously for 90 min, followed by turning off the lamp and recording temperature data for an additional 15 min. Temperature measurements were extracted as mean values from the central regions of the electrodes of each sample.

##### Statistical Analysis

All data are reported as mean ± standard deviation (SD). SD values were calculated using the STDEV.P function in Microsoft Excel. No data transformation, normalization, or outlier exclusion was applied, and no statistical hypothesis testing was performed. Data processing and visualization were carried out using Microsoft Excel and Origin.

Optical measurements were performed on three different spots of each sample (*n* = 3), and the averaged values were reported with SD. Photovoltaic parameters (*J*_sc_, *V*_oc_, FF, and PCE) were extracted from the *J*–*V* curves of five independently fabricated devices per condition (*n* = 5). Reported values represent the mean ± SD to indicate device‐to‐device variability. EQE measurements were performed on the same PSC before and after composite attachment. For each condition, three repeated measurements were collected (*n* = 3). In the graphs (Figure 4f,g), solid lines represent the average of the measurements, while shaded regions indicate ±SD. Integrated *J*_sc_ values were calculated from these averaged EQE spectra.

## Supporting Information

Supporting Information is available from the Wiley Online Library or from the author.

## Conflict of Interest

The authors declare no conflict of interest.

## Supporting information

Supplementary Material

## Data Availability

The data that support the findings of this study are available from the corresponding author upon reasonable request.
