# Supplementary material for: Enhancing the Performance and Photostability of Perovskite Solar Cells with a Multifunctional Light‐Management Composite
Source: Small Sci. 2025 Oct 2;5(12):e202500330. doi: 10.1002/smsc.202500330 (PMC12697776; doi:10.1002/smsc.202500330)
Supplement: Supplementary file 1 — Supplementary Material [file SMSC-5-e202500330-s001.pdf]

Supporting information

Enhancing the Performance and Photostability of Perovskite Solar Cells with a Multifunctional Light-Management Composite

Seyede Maryam Mousavi[a], Mostafa Othman[b], Fanxing Zou[a], Noora Lamminen[c], Girish Tewari[a], Hossein Baniasadi[d], Pedro Silva[a], Yujiao Dong[a], Janne Halme[e], Aïcha Hessler-Wyser[b], Christian M. Wolff[b], Paola Vivo[c], Muhammad Imran Asghar[f], Jaana Vapaavuori[a]

[a]Department of Chemistry and Materials Science, Aalto University School of Chemical Engineering, Espoo, Finland
[b] Institute of Electrical and Microengineering (IEM) Photovoltaics and Thin-Film Electronics Laboratory (PV-Lab), École Polytechnique Fédérale de Lausanne (EPFL), Neuchâtel 2000, Switzerland
[c] Hybrid Solar Cells, Faculty of Engineering and Natural Sciences, Tampere University, P.O. Box 541, Tampere, FI-33014 Finland
[d] Polymer Synthesis Technology, School of Chemical Engineering, Aalto University School of Science, Espoo, Finland
[e] Department of Applied Physics, Aalto University School of Science, Espoo, Finland
[f] Renewable Energy Technologies Group, Faculty of Engineering and Natural Sciences, Tampere University, P.O. Box 541, Tampere, FI-33014 Finland

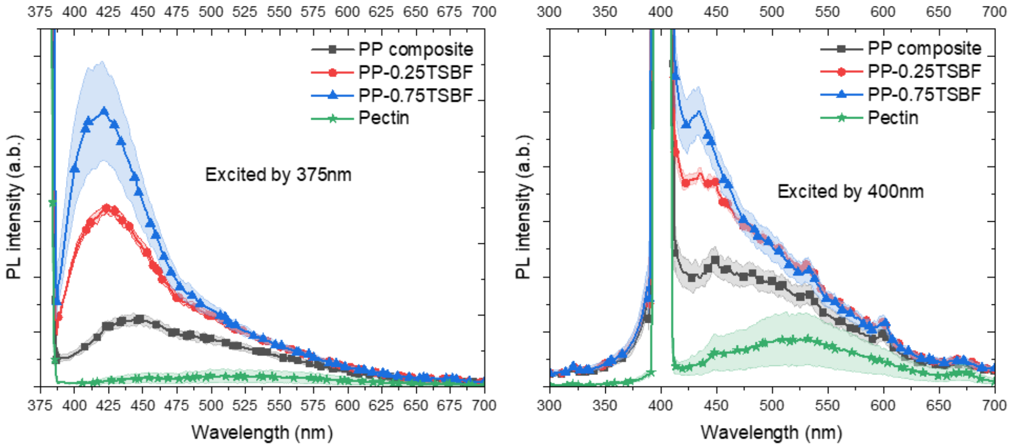

Figure S1 The PL emission from the PP composite, PP-0.25TSBF and PP-0.75TSBF composites, excited with 375 nm (on the left) and 400nm (on the right).

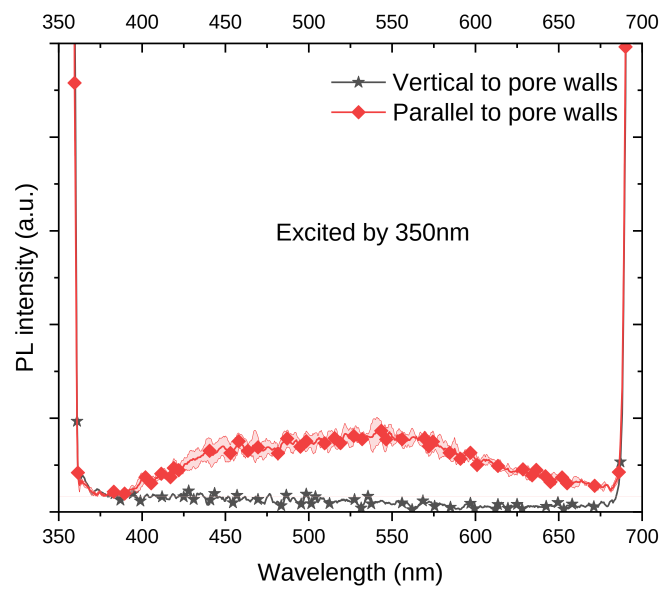

Figure S2 The PL emission from pectin cryogels in different orientations, parallel to pore walls and vertical to pore walls. The test was performed with the excitation wavelength of 350 nm

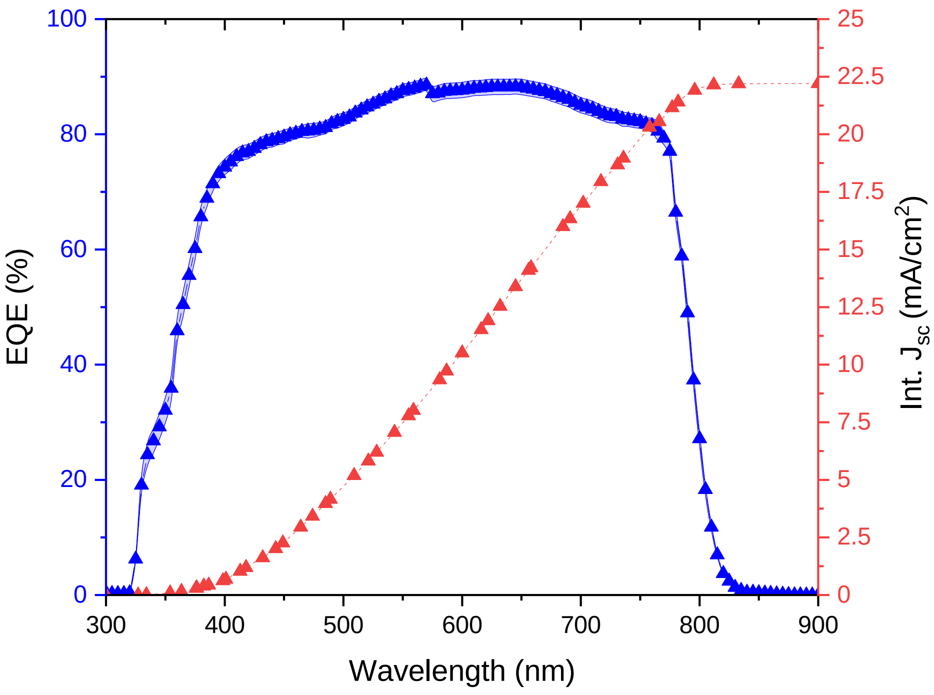

Figure S3 The EQE and integrated current of the PP-0.75TSBF integrated PSCs

Table S1 The photovoltaic parameters of the PSCs before and after attaching the composites

PSCs		J_{sc} (mA/cm^2)	V_{oc} (v)	*FF*	PCE (%)
PP composite	Before	22.7±0.2	0.98	0.7	16.1±0.3
	After	23.2±0.3	0.98	0.7	16±0.7
PP-0.25TSBF	Before	22.7±0.5	0.99	0.71	16.1
	After	23.7±0.1	0.99	0.71	16.9±0.4

Table S2 The J_{sc} recorded from J-V scans and calculated from the EQE signals of the PSCs before and after attaching the composites

PSCs		**J_{sc} (mA/cm^2) From *J-V* scan**		**Int J_{sc} (mA/cm^2) From EQE**
PP composite	Before	22.7±0.2	Before	21.9
	After	23.2±0.3	After	22.05
PP-0.25TSBF	Before	22.7±0.5	Before	22.5
	After	23.7±0.1	After	23.5

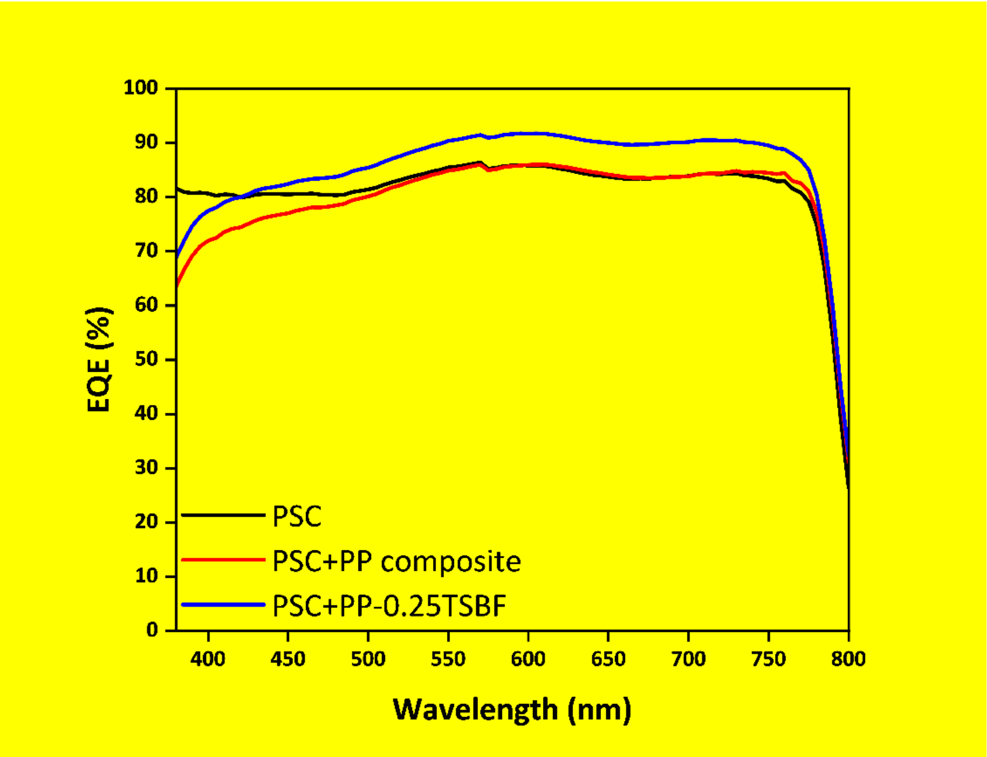

Figures S4 EQE spectra for the pristine PSC, PP composite-integrated PSC, and 0.25TSBF- PP composite PSC.

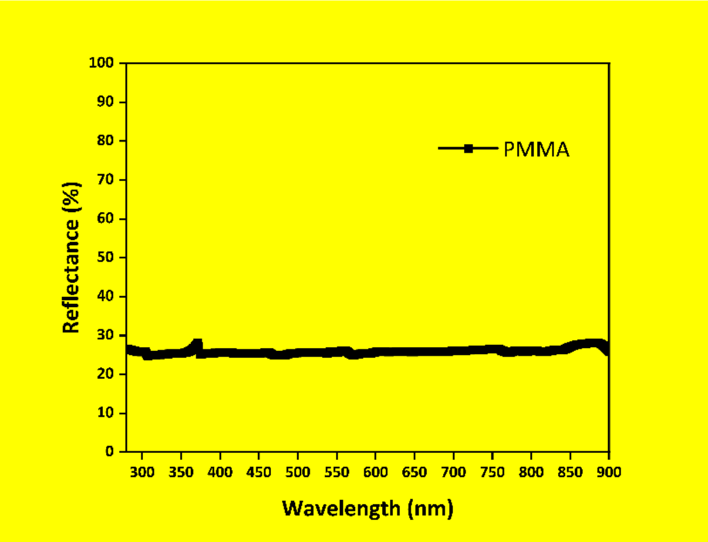

Figure S5 The reflection from the surface of a standalone PMMA film

Table S3 The initial photovoltaic parameter of the n-i-p PSCs fabricated for the MPPT test.

PSCs	PCE (%)	Jsc (mA/cm2)	Voc (v)	FF
PSC	14.35	20	1.09	0.66
PSC+PP composite	12.7	18.1	1.08	0.67
PSC+PP-0.25TSBF	13.8	13.5	1.08	0.66
PSC+PP-0.75TSBF	13	17.7	1.08	0.67

It should be noted that all devices were measured without shadow masks, as the added films created a height difference that would have caused shading effects. Consequently, the reported values in Table S3 may have slightly lower accuracy compared to measurements performed with shadow masks for the pin devices that were used for the performance measurements.
